# Factors associated with underrepresented minority physician scientist trainee career choices

**DOI:** 10.1186/s12909-020-02328-6

**Published:** 2020-11-11

**Authors:** Aisha L. Siebert, Shinnyi Chou, Omar Toubat, Alexander J. Adami, Hajwa Kim, Dania Daye, Jennifer M. Kwan

**Affiliations:** 1 Northwestern University, Chicago, USA; 2 University of Pittsburgh, Pittsburgh, USA; 3 Keck School of Medicine of USC, University of Southern California, Los Angeles, USA; 4 Department of Internal Medicine, University of Washington, Seattle, WA USA; 5 University of Illinois, Chicago, IL USA; 6 Massachusetts General Hospital, Boston, USA; 7 Section of Cardiovascular Medicine, Yale University School of Medicine, New Haven, CT 06511 USA

## Abstract

**Background:**

Recently, there have been concerted efforts to improve racial and ethnic diversity in the physician-scientist workforce. Identifying factors associated with career choices among those underrepresented in medicine and science is a necessary first step to advance this objective. The aim of the present study was to assess the attitudes and factors associated with academic and research career interests among underrepresented predoctoral physician-scientists.

**Methods:**

A cross-sectional 70-question survey was distributed to all predoctoral single degree (MD or DO) and dual degree (MD/PhD or DO/PhD) trainees at 32 medical schools in the United States from 2012 to 2014. Main outcomes included factors important to advancement in academic medicine, intended medical specialty, and future career plans. To test the post-hoc hypothesis of whether trainees from underrepresented groups have differing perceptions of career trajectories and obstacles than their counterparts, we evaluated responses according to self-identified race/ethnic status using Chi-square and Fisher’s exact tests. All tests were two-sided and significance level of < 0.05 was used.

**Results:**

There were a total of 4433 responses representing all predoctoral training stages. The response rate was 27%. Most respondents were single degree trainees (MD/DO 79% vs MD/DO-PhD 21%). Most respondents self-identified as White (67%), followed by Multi-racial or Other (14.3%), Asian or Pacific Islander (10.4%), Hispanic (6%), and Black or African American (4.1%). Desired career sector, career intention, and clinical specialty interest differed across race/ethnic groups. With respect to career selection factors, anticipated non-work related responsibilities during residency were also significantly different between these groups. By multivariable regression analysis, Black or African American trainees were significantly less likely than White trainees to indicate a career in academia (OR 0.496, 95% CI 0.322–0.764) and basic research (OR 0.314, 95% CI 0.115–0.857), while Multi-racial or Other trainees were also less likely than White trainees to indicate a career in academia (OR 0.763, 95% CI 0.594–0.980).

**Conclusions:**

These data represent the first in-depth survey of career aspirations, perceptions, and interests between demographically underrepresented and non-underrepresented predoctoral physician-scientist trainees. Our results identify key differences between these cohorts, which may guide efforts to improve diversity within the physician-scientist workforce.

**Supplementary Information:**

The online version contains supplementary material available at 10.1186/s12909-020-02328-6.

## Background

Recently, there have been significant efforts aimed at improving racial and ethnic diversity in physician-scientist training programs and the academic biomedical workforce. Organizations such as the National Institutes of Health (NIH) and the Association of American Medical Colleges (AAMC) have defined underrepresented individuals in medicine (URM) as those who self-identify with racial and ethnic groups that are underrepresented in medicine relative to their prevalence in the general population [1, 2]. In 2014, the National Institutes of Health (NIH) Physician-Scientist Workforce Working Group Report identified the need to improve diversity as a principal recommendation for the NIH and other organizations dedicated to training the next generation of physician-scientists [3]. Their analysis not only demonstrated that the number of MD/PhD applicants identifying as African American, Native American, or Hispanic have been stagnant for several years, but also that physician-scientists from these groups have been persistently disproportionately underrepresented among NIH funded investigators. In particular, it was observed that physician-scientist investigators from these demographic groups comprised only 7% of the total research project grant applicant pool and 4.7% of awardees [3].

While there are several potential explanations for this disconcerting trend, factors such as opportunities for mentorship, clinical specialty decisions, and structural or implicit bias have all been implicated. Specifically, previous studies have described that URM faculty self-report experiencing less direct mentorship and greater discrimination by superiors or colleagues [4–7]. In addition, prior analyses have shown that even after controlling for academic productivity, URM faculty remain less likely to be promoted and more likely to hold part-time faculty positions than their non-URM peers, suggesting that there may be residual implicit bias in the academic environment and career advancement process [8–10]. While addressing these issues is crucial for improving success among later stage URM physician-scienists, equally important to the effort of improving diversity in the physician-scientist workforce is understanding the intended career choices and obstacles of URM trainees entering the physician-scientist training pipeline. Despite the clear importance of understanding the perspectives of predoctoral URM physician-scientist trainees, there are currently no nationally representative data focusing on this topic.

The American Physician Scientists Association (APSA) is a national, trainee-led organization that was started with the goal of advocating for training and career development opportunities for future physician-scientists [11]. In this role, APSA had led multiple national cohort surveys assessing career interests and attitudes among physician-scientist trainees [12, 13]. The primary objective of the present study was to evaluate specialty interest, intended career trajectory, and obstacles in a nationally representative cohort of predoctoral physician-scientist trainees, with a particular focus on understanding the factors associated with academic and research career interests among URM trainees. We hypothesized that URM and non-URM trainees differ in academic and research interests and perceive unique career selection factors and obstacles.

## Methods

### Study design

This study is a cross-sectional survey of predoctoral trainees enrolled in single degree (MD, DO) or dual degree (MD/PhD, DO/PhD) training programs at academic medical institutions in the United States. The main objective of the study was to examine whether there are racial/ethnic differences in career selection factors, perceived research interest, intended medical specialty, experienced and anticipated obstacles, and future career plans in this cohort. The study protocol was reviewed and exempted by the Institutional Review Board at the University of Illinois at Chicago and the University of Pennsylvania. To ensure unique responses, participants submitted school email addresses, which were later de-identified. MD-PhD trainees were those enrolled in tuition sponsorship by MD-PhD programs. MD-RI status was defined by a self-reported career interest of at least 50% research among single degree candidates. This minimum research interest was selected because 50% is often the minimum full time effort commitment required for mentored NIH career development awards [14]. All demographic characteristics, including race and ethnicity, were self-defined by respondents. To assess the generalizability of responses from this study cohort, we compared demographics and clinical specialty interests between survey respondents with those of the Association of American Medical College’s (AAMC’s) enrollment and graduating medical student databases [15]. To facilitate a comparison of specialty interests, we.

categorized specialties into the following groups: primary care/medicine, surgical, acute care, diagnostic, and undecided/other (Supplemental Tables 1 and 2).

### Survey tool

A 70-question survey was previously developed and validated at the University of Illinois at Chicago to assess factors important to influencing MD and MD-PhD student interest in research careers (Supplementary material) [12]. For the present study, the survey was administered to all medical students at 32 nationally representative medical schools from 2012 through 2014. According to the AAMC annual report on the total number of matriculants at each of the 32 institutions in the year the survey was first administered, the target population for this survey was 16,418 trainees [15]. Three reminder emails were sent throughout this collection period, averaging one reminder every 8 months. Students were targeted by email through student list serves, members of the American Medical Women’s Association, as well as through institutional representatives of the American Physician Scientists Association (APSA). Institutions were selected with the intention of balancing geographic distribution, funding status (public vs private), and NIH-funded MSTP program representation. The primary criterion for entry was that the student was enrolled in either the single degree (MD, DO) or dual degree (MD/PhD, DO/PhD) track at one of the 32 targeted institutions at the time of survey completion. Participants consented to join the study on the first page of the survey link and were given the option to remove their consent and withdraw participation at any time during the survey. A more complete analysis of career intentions in the entire cohort comparing differences between single and dual-degree trainees was completed and previously published [13]. Data were collected using an online survey tool (SurveyMonkey, www.surveymonkey.com).

### Statistical methods

The survey questions were developed by physician-scientist trainees with feedback from a survey design team and faculty at the University of Illinois at Chicago. Some questions included in this survey were from previously published instruments for graduate and professional student socialization, with permission from the author [16]. The survey consisted of multiple choice and ordinal scale questions developed specifically to permit exploration of differences in the specific aspirations and experiences reported by various respondent characteristics. Assessment of the face validity of items in this questionnaire was conducted by experts in the field as described in the pilot analysis [12]. Chi-squared tests were used to measure associations between categorical variables. Where data violated minimum expected cell counts, Fisher’s exact test was performed. Logistic regression was used to determine the unique influence of each predictor variable on the intention to go into academic medicine, and other outcomes such as basic science research, translational research, and clinical research after residency, controlling for other variables in the model: age, sex, ethnicity, training stage, specialty intention, ability to identify a mentor, how medical school was paid for. All tests were performed using SPSS v16. All tests of significance were 2-sided and a *p*-value less than 0.05 was considered statistically significant.

## Results

### Demographics

There was a total of 4433 responses, yielding an estimated overall response rate of 27%. Of these, 4033 specified their race/ethnicity and were included in this analysis. There were 2566 (63.6%) MD trainees, 639 (15.8%) single-degree trainees interested in pursuing research intense careers (MD-RI), and 828 (20.5%) dual-degree MD/PhD trainees. While the majority of all respondents were in the first 2 years of medical school (53.5%), all medical and graduate level training stages were represented (Table 1). The majority of respondents self-identified as being White (71%), followed by Multiracial/Other (14.3%), Asian (10.5%), Hispanic (6.0%), and Black/African American (4.1%). A similar demographic trend was observed when stratifying trainees according to training paradigm (MD vs MD-RI vs MD/PhD) (Table 1). Compared to the 2014 AAMC data on the racial/ethnic identities among medical students, respondents to this survey were more likely to be White (71% vs 55.7%) and Multiracial/Other (14.3% vs 7.2%), and less likely to be Asian (10.6% vs 23.3%). The proportion of Black/African American (4.1% vs 5.3%) and Hispanic (6.0% vs 4.7%) survey respondents were similar to national estimates provided by the AAMC. A complete list of demographic characteristics is summarized in Table 1.

**Table 1:** Demographic characteristics of respondents by race/ethnicity

Demographic	Total n (%)	Asian or Pacific Islander n (%)	Black or African American n (%)	Multiracial or Other n (%)	White, n (%)	***P***-value	Hispanic n (%)	***P***-value (Hispanic)
Training pathway						**<.0001**		**0.008**
MD-RI	639 (15.84%)	89 (20.84%)	35 (20.1%)	141 (24.4%)	374 (13.1%)		56 (23.1%)	
MD-PHD	828 (20.5%)	101 (23.7%)	30 (18.0%)	105 (18.2%)	592 (20.7%)		45 (18.6%)	
MD	2566 (63.63%)	237 (55.5%)	102 (61.1%)	331 (57.4%)	1869 (66.3%)		141 (58.3%)	
Total	4033 (100%)	427 (100%)	167 (100%)	577 (100%)	2862 (100%)		242 (100%)	
Gender	0.0034		0.2158					
Female	2269 (56.5%)	259 (61.8%)	114 (68.7%)	309 (53.8%)	1587 (55.6%)		133 (55.4%)	
Male	1736 (43.2%)	159 (38.0%)	52 (31.3%)	262 (45.6%)	1263 (44.2%)		105 (43.8)	
Other	10 (0.25%)	1 (0.24%)	0 (−-)	3 (0.52%)	6 (0.21%)		2 (0.83%)	
TOTAL	4015 (100%)	419 (100%)	166 (100%)	574 (100%)	2856 (71.1%)		240 (100%)	
Training stage						**0.0108**		0.1864
Medical School Year 1	1131 (28.2%)	128 (30.2%)	46 (27.7%)	179 (31.1%)	778 (27.3%)		84 (35.0%)	
Medical School Year 2	1015 (25.3%)	115 (27.1%)	35 (21.1%)	156 (27.1%)	709 (24.9%)		65 (27.1%)	
Medical School Year 3	655 (16.6%)	65 (15.3%)	29 (17.5%)	76 (13.2%)	485 (17.5%)		33 (13.8%)	
Medical School Year 4	666 (16.6%)	53 (12.5%0	32 (19.3%)	93 (14.4%)	498 (17.5%)		34 (14.2%)	
Graduate School Year 1	127 (3.16%)	8 (1.89%)	7 (4.22%)	18 (3.13%)	94 (3.30%)		8 (3.3%)	
Graduate School Year 2	102 (2.54%)	12 (2.83%)	3 (1.81%)	16 (2.78%)	71 (2.49%)		3 (1.3%)	
Graduate School Year 3	84 (2.09%)	13 (3.07%)	3 (1.81%)	7 (1.22%0	61 (2.14%)		1 (0.4%)	
Graduate School Year 4	85 (2.12%)	10 (2.36%)	2 (1.20%)	12 (2.08%)	18 (0.63%)		3 (1.3%)	
Graduate School Year 5 or more	34 (0.84%)	4 (0.94%)	1 (0.60%)	2 (0.35%)	27 (0.95%)		0 (0.0%)	
TOTAL	4018 (100%)	424 (100%)	166 (100%)	576 (100%)	2852 (100%)		240 (100%)	
Marital Status						**<.0001**		0.0595
Is married/partnered	1033 (26.2%)	855 (30.6%)	62 (14.8%)	21 (13.0%)	95 (16.8%)		50 (20.8%)	
Is NOT married/partnered	2911 (73.8%)	357 (85.2%)	140 (87.0%)	472 (83.3%)	1942 (69.4%)		190 (79.2%)	
TOTAL	3944 (100%)	419 (100%)	161 (100%)	567 (100%)	2797(100%)		240 (100%)	
Parental Status	<.0001		0.6096					
Has a child/children	225 (5.71%)	4 (0.95%)	11 (6.79%)	23 (4.06%)	187 (6.69%)		12 (5.0%)	
Does NOT have a child/children	3718 (94.3%)	415 (99.1%)	151 (93.2%)	543 (95.9%)	2609 (93.3%)		229 (95.0%)	
TOTAL	3943 (100%)	419 (100%)	162 (100%)	566 (100%)	2796 (100%)		241 (100%)	
Advanced degree of mother^1^								
MD or DO	330 (8.18%)	48 (11.2%)	7 (4.19%)	77 (13.3%)	198 (6.92%)	**<.0001**	13 (5.37%)	0.1120
DDS	17 (0.42%)	2 (0.47%)	1 (0.60%)	3 (0.52%)	11 (0.38%)	0.9454	0 (0.00%)	0.3016
PhD	208 (5.16%)	35 (8.2%)	11 (6.59%)	34 (5.89%)	128 (4.47%)	**0.0069**	7 (2.89%)	0.0981
DVM	9 (0.22%)	0 (−-)	0 (−-)	0 (−-)	9 (0.31%)	0.2969	0 (−-)	0.5791^a^
Master’s	998 (24.8%)	120 (28.1%)	42 (25.2%)	143 (24.8%)	693 (24.2%)	0.3864	47 (19.4%)	0.0558
Area of medicine mother works in^1^								
Academia	13 (3.04%)	2 (1.20%)	18 (3.12%)	70 (2.45%)	103 (2.55%)	0.4711	2 (0.83%)	0.0851
Private practice	158 (3.92%)	16 (3.75%)	3 (1.80%)	40 (6.93%)	99 (3.46%)	**0.0005**	4 (1.65%)	0.0657
Consulting	6 (0.15%)	0 (−-)	0 (−-)	0 (−-)	6 (0.15%)	0.4828	1 (0.41%)	0.2610^a^
Industry	12 (0.30%)	4 (0.94%)	0 (−-)	1 (0.17%)	7 (0.24%)	0.0735	1 (0.41%)	0.3630^a^
Advanced degree of father^1^								
MD or DO	669 (16.6%)	75 (17.6%)	19 (11.4%)	111 (19.2%)	464 (16.2%)	0.0789	33 (13.6%)	0.2296
DDS	51 (1.26%)	5 (1.17%)	2 (1.20%)	10 (1.73%)	34 (1.19%)	0.7566	4 (1.65%)	0.1743^a^
PhD	512 (12.7%)	110 (25.8%)	18 (10.8%)	106 (18.4%)	278 (9.71%)	**<.0001**	10 (4.13%)	**<.0001**
JD	205 (5.08%)	2 (0.47%)	2 (1.20%)	15 (2.60%)	186 (6.50%)	**<.0001**	9 (3.72%)	0.3275
DVM	20 (0.50%)	0 (−-)	0 (−-)	1 (0.17%)	19 (0.66%)	0.1210	0 (−-)	0.2965^a^
Master’s	952 (23.6%)	118 (27.6%)	39 (23.4%)	159 (27.6%)	636 (22.2%)	**0.0078**	41 (16.9%)	0.0139
Area of medicine father works in^1^								
Academia	194 (4.81%0	22 (5.15%)	2 (1.20%)	37 (6.41%)	133 (4.65%)	**0.0407**	10 (4.13%)	0.6337
Private practice	414 (10.3%)	40 (9.37%)	15 (8.98%)	71 (12.3%)	288 (10.1%)	0.3332	21 (8.68%)	0.4236
Consulting	31 (0.77%)	2 (0.47%)	0 (−-)	3 (0.52%)	26 (0.91%)	0.3917	2 (0.83%)	0.2796^a^
Industry	33 (0.82%)	6 (1.41%)	0 (−-)	2 (0.35%0	25 (0.87%)	0.1809	1 (0.41%)	0.2790^a^
How primarily paid for medical school^1^ n (% of 4072)						**<.0001**		**<.0001**
MD-PhD or DO-PhD sponsored	726 (18.28%)	91 (21.7%)	25 (15.4%)	97 (17.0%)	513 (18.2%)		38 (15.77%)	
Scholarships	378 (9.52%)	43 (10.3%)	48 (29.6%)	68 (11.9%)	219 (7.77%)		34 (14.11%)	
Grants	71 (1.79%)	13 (3.10%)	6 (3.70%)	15 (2.63%)	37 (1.31%)		15 (6.22%)	
Loans	2076 (52.3%)	163 (38.9%)	77 (47.5%)	243 (42.6%)	1593 (56.5%)		135 (56.02%)	
National Service	48 (1.21%)	1 (0.24%)	1 (0.62%)	9 (1.58%)	37 (1.31%)		3 (1.24%)	
Personal Savings	45 (1.13%)	6 (1.43%)	0 (−-)	5 (0.88%)	34 (1.21%)		2 (0.83%)	
Family/partner Support	619 (15.6%)	102 (24.3%)	4 (2.47%)	133 (23.3%)	380 (13.5%)		14 (5.81%)	
Work	5 (0.15%)	0 (−-)	0 (−-)	0 (−-)	6 (0.15%)		0 (−-)	
Other	2 (0.05%)	0 (−-)	1 (0.62%)	0 (−-)	1 (0.04%)		0 (−-)	

^a^ Fisher’s Exact calculated due to minimum cell count violations

^1^ Respondents could select all applicable choices, will not sum to 100%

### Familial characteristics and financial considerations

Familial characteristics varied significantly across racial and ethnic groups. Rates of marriage/partnerships were found to be statistically significantly different among respondents, with Asian trainees most likely to be married/partnered (30.6%), followed by Hispanic (20.8%), White (16.8%), Black/African American (14.8%), and Multiracial/Other respondents (13.0%) (Table 1). The proportion of trainees with children demonstrated a different trend, with Black/African American trainees most likely to have children (6.8%), followed by White (6.7%), Hispanic (5.0%), Multiracial/Other (4.1%), and Asian trainees (1.0%) (Table 1).

Racial and ethnic differences were also observed with respect to parental educational attainment and methods for financing medical school (Table 1). The majority of Asian trainees (62.8%) reported having a father and/or mother with an MD/DO or PhD degree. This was followed by Multiracial/Other (56.8%), White (37.3%), Black/African American (33.0%), and Hispanic (26.0%) trainees. The most common mechanism of paying for medical school tuition for all racial/ethnic groups was loans. However, the second most frequently selected response differed across these groups, with Asians (24.3%) and Multiracial/Others (23.3%) indicating family/partner support, Whites (18.2%) and Hispanics (15.8%) indicating MD/PhD or DO/PhD sponsorship, and Black/African American trainees (29.6%) indicating scholarships.

### Career intentions and specialty interest

We then compared career sector, career intention, and clinical specialty interests (Table 2). Trainees from all racial/ethnic groups listed academia as the primary career sector of interest (Asian 49.4%; Black/African American 40.8%; Multiracial/Other 50.5%; White 46.4%; Hispanic 49.4%). However, secondary and tertiary options were found to split across these groups. Asian and Black/African American trainees expressed interest in hospitalist (Asian 34.5%; Black/African American 25.5%) then private practice careers (Asian 19.4%; Black/African American 22.9%), whereas Multiracial/Other, White, and Hispanic trainees preferred private practice (Multiracial/Other 20.4%; White 28.6%; Hispanic 24.6%) over hospitalist careers (Multiracial/Other 18.8%; White 16.1%; Hispanic 19.5%).

**Table 2:** Career sector, career intentions, and clinical specialty interests

	Total n (%)	Asian or Pacific Islander n (%)	Black or African American n (%)	Multiracial or Other n (%)	White n (%)	***P***-value	Hispanic n (%)	***P***-value (Hispanic)
Sector^1^						**<.0001**		0.5220
Consulting	71 (1.84%)	13 (3.15%)	3 (1.91%)	10 (1.82%)	45 (1.65%)		7 (2.97%)	
Academia	1812 (47.1%)	204 (49.4%)	64 (40.8%)	277 (50.5%)	1267 (46.4%)		103 (49.36%)	
Industry	66 (1.71%)	2 (0.48%)	3 (1.91%)	11 (2.00%)	50 (1.83%)		5 (2.12%)	
Government	109 (2.83%)	5 (1.215)	5 (3.18%)	23 (4.19%)	76 (2.78%)		11 (4.66%)	
Private Practice	1009 (26.2%)	80 (19.4%)	36 (22.9%)	112 (20.4%)	781 (28.6%)		58 (24.58%)	
Hospitalist	683 (17.7%)	101 (24.5%)	40 (25.5%)	103 (18.8%)	439 (16.1%)		46 (19.49%)	
Other	34 (0.88%)	1 (0.24%)	3 (1.91%)	4 (0.73%)	26 (0.95%)		3 (1.27%)	
N/A	20 (0.52%)	3 (0.73%)	1 (0.64%)	1 (0.18%)	15 (0.55%)		1 (0.42%)	
Nonprofit	20 (0.52%0	0 (−-)	1 (0.64%)	3 (0.55%0	16 (0.59%)		0 (−-)	
Community Hospital	27 (0.70%)	4 (0.97%)	1 (0.64%)	5 (0.91%)	17 (0.62%)		2 (0.85%)	
Career Intention^1^						**0.0005**		0.5440
Education	225 (5.85%)	24 (5.83%)	12 (7.79%)	29 (5.33%)	160 (5.85%)		9 (3.81%)	
Basic research	197 (5.12%)	28 (6.80%)	1 (0.65%)	26 (4.78%)	142 (5.19%)		15 (6.36%)	
Clinical research	199 (5.185)	26 (6.31%)	13 (8.44%)	37 (6.80%)	123 (4.50%)		16 (6.78%)	
Translational research	435 (11.3%)	52 (12.3%)	21 (13.6%)	71 (13.1%)	291 (10.6%)		25 (10.59%)	
Clinical duties	2503 (65.1%)	241 (58.5%)	87 (56.5%)	324 (59.7%)	1851 (67.7%)		149 (63.14%)	
Therapeutics/diagnostic	69 (1.80%)	11 (2.67%)	2 (1.30%)	8 (1.47%)	48 (1.76%)		4 (1.69%)	
Advocacy	91 (2.37%)	9 (2.18%)	10 (6.49%)	20 (3.68%)	52 (1.90%)		9 (3.81%)	
Administration	56 (1.46%)	12 (2.91%)	5 (3.25%)	16 (2.94%)	23 (0.84%)		5 (2.12%)	
Other	51 (1.33%)	7 (1.70%)	1 (0.65%)	8 (1.47%)	35 (1.28%)		2 (0.85%)	
Specialty^2^						**< 0.01**		0.1632
Allergy and Immunology	16 (0.42%)	2 (0.49%)	0 (−-)	1 (0.18%)	13 (0.48%)		1 (0.43%)	
Anesthesiology	151 (3.93%)	18 (4.43%)	10 (6.41%)	21 (3.80%)	102 (3.75%)		5 (2.17%)	
Colon and Rectal Surgery	1 (0.03%)	0 (−-)	1 (0.03%)	0 (−-)	0 (−-)		0 (−-)	
Dermatology	94 (2.45%)	9 (2.22%)	4 (2.56%)	16 (2.89%)	65 (2.39%)		6 (2.61%)	
Emergency Medicine	320 (8.34%)	22 (5.42%)	10 (6.41%)	29 (5.24%)	259 (9.51%)		17 (7.39%)	
Family Medicine	285 (7.43%)	22 (5.42%)	5 (3.21%)	29 (5.24%)	229 (8.41%)		21 (9.13%)	
Internal Medicine	351 (9.15%)	44 (10.8%)	14 (8.97%)	52 (9.40%)	241 (8.85%)		15 (6.52%)	
Internal Medicine – Cardiology	135 (3.52%)	17 (4.19%)	9 (5.77%)	40 (7.23%)	69 (2.53%)		8 (3.48%)	
Internal Medicine – Endocrinology	21 (0.83%)	2 (0.49%)	0 (−-)	8 (1.45%)	22 (0.81%)		2 (0.87%)	
Internal Medicine – Gastroenterology	40 (1.04%)	6 (1.48%)	2 (1.28%)	4 (0.72%)	28 (1.03%)		2 (0.87%)	
Internal Medicine —Hematology/Oncology	184 (4.79%)	35 (8.62%)	3 (1.92%)	21 (3.80%)	125 (4.59%)		8 (3.48%)	
Internal Medicine – Infectious Disease	103 (2.68%)	6 (1.48%)	5 (3.21%)	10(1.81%)	82 (3.01%)		7 (3.04%)	
Internal Medicine – Pulmonology	19 (0.50%)	1 (0.25%)	0 (−-)	3 (0.54%)	4(0.15%)		0 (−-)	
Internal Medicine – Rheumatology	8 (0.21%)	1 (0.25%0	0 (−-)	3 (0.54%0	4 (0.15%)		2 (0.87%)	
Medical Genetics	18 (0.47%)	2 (0.49%)	0 (−-)	1 (0.18%)	15 (0.55%)		1 (0.43%)	
Neurological Surgery	81 (2.11%)	9 (2.22%)	4 (2.56%)	15 (2.71%)	53 (1.95%)		6 (2.61%)	
Neurology	170 (4.43%)	18 (4.43%)	2 (1.28%)	27 (4.88%)	123 (4.52%)		11 (4.78%)	
Nuclear Medicine	3 (0.08%)	0 (−-)	0 (−-)	0 (−-)	3 (0.11%)		0 (−-)	
Obstetrics and Gynecology	180 (4.69%)	14 (3.45%)	11 (7.05%)	25 (4.52%)	130 (4.77%)		16 (6.96%)	
Ophthalmology	114 (2.97%)	21 (5.17%)	0 (−-)	28 (5.06%)	65 (2.39%)		5 (2.17%)	
Orthopedic Surgery	147 (3.83%)	14 (3.45%)	5 (3.21%)	25 (4.52%)	103 (3.78%)		15 (6.52%)	
Otolaryngology	79 (2.06%)	7 (1.72%)	1 (0.64%)	18 (3.25%)	53 (1.95%)		3 (1.30%)	
Pathology	56 (1.46%)	6 (1.48%)	3 (1.92%)	6 (1.08%)	41 (1.51%)		1 (0.43%)	
Pediatrics	495 (12.90%)	47 (11.58%)	26 (16.7%)	58 (10.5%)	364 (13.4%)		27 (11.74%)	
Physical Medicine and Rehabilitation	35 (0.91%)	2 (0.49%)	2 (1.28%)	7 (.1.27%)	24 (0.88%)		3 (1.30%)	
Plastic Surgery	40 (1.04%)	5 (1.23%)	1 (0.64%)	2 (0.36%)	32 (1.18%)		2 (0.87%)	
Preventative Medicine	12 (0.31%)	5 (1.23%)	1 (0.64%)	3 (0.54%)	3 (0.11%)		1 (0.43%)	
Psychiatry	114 (2.97%)	7 (1.72%)	8 (5.13%)	14 (2.53%)	85 (3.12%)		4 (1.74%)	
Radiation Oncology	54 (1.41%)	13 (3.20%)	5 (3.21%)	6 (1.08%)	30 (1.10%)		1 (0.43%)	
Radiology	107 (2.79%)	9 (2.22%)	4 (2.56%)	22 (3.98%)	72 (2.64%)		2 (0.87%)	
Surgery	237(6.18%)	23 (5.67%)	12 (7.69%)	34 (6.15%)	168 (6.17%)		21 (9.13%)	
Thoracic Surgery	31 (0.81%)	1 (0.25%0	1 (0.64%)	8 (1.45%)	21 (0.77%)		3 (1.30%)	
Urology	41 (1.07%)	5 (1.23%)	2 (1.28%)	8 (1.45%)	26 (0.95%)		5 (2.17%)	
Other	85 (2.21%)	13 (3.20%)	5 (3.21%)	11 (1.99%)	56 (2.06%)		9 (3.91%)	

^a^ Fisher’s Exact calculated due to minimum cell count violations

^1^ Respondents could select all applicable choices, will not sum to 100%

^2^ Respondents could select up to TWO choices, will not sum to 100%

^3^ Respondents could select up to THREE choices, will not sum to 100%

With respect to clinical duties, basic/translational research, and clinical research career intentions, there were racial/ethnic differences in the responses across each of these domains (Table 2). White trainees were most likely to indicate wanting to pursue clinical duties (67.6%) followed by Hispanic (63.1%), Multiracial/Other (59.7%), Asian (58.5%), and Black/African American (56.5%) trainees. For basic/translational research, Asian trainees were the most interested in pursuing this career intention (19.1%) followed by Multiracial/Other (17.9%), Hispanic (17.0%), White (15.8%), and Black/African American (14.3%) trainees. Trends in clinical research interest differed, with Black/African American trainees expressing the greatest interest in this career practice (8.4%) followed by Hispanic (6.8%), Multiracial/Other (6.8%), Asian (6.3%), and White (4.5%) trainees. Black/African American trainees were also most likely to express interest in education and advocacy (Table 2).

Primary care (internal medicine, pediatrics, and family medicine) ranked among the top specialty intentions for all trainees (Table 2). Between primary care specialties, trainees from all racial/ethnic groups preferred pediatrics over internal medicine and family medicine. With regard to general surgery, Hispanics were the most interested in pursuing this specialty (9.1%) followed by Black/African American (7.7%), White (6.2%), Multiracial/Other (6.2%), and Asian (5.7%) trainees. The remaining responses for acute care, diagnostic, medical, and surgical sub-specialties are listed in Table 2. To assess generalizability of these trends in specialty interest, we compared our survey responses to those of trainees surveyed in the 2014 AAMC Graduating Student Questionnaire (Supplementary Table 3). When analyzing specialty interest according to pre-specified specialty categories, both our survey (*p* = 0.001) and that of the AAMC (*p* = 0.01) show that specialty interests differ significantly across racial/ethnic groups (Supplementary Tables 1 and 2).

To assess the association between race/ethnicity and intended career path (i.e. academia, basic research, translational research or clinical research) a logistic regression model was used, adjusting for sex, age, training stage, access to mentors, specialty intention, and how medical school was paid for. Asians were significantly more likely to report interest in an academic career (OR 1.69, 95 CI 1.03–2.78), and intention to do basic research (OR 3.229, 95 CI 1.123–9.28) compared to Black/African American trainees (Table 3). Black/African American trainees were less likely to have career intentions in academia (OR 0.49, 95 CI 0.322–0.764) and basic research (OR 0.314 95 CI 0.115–0.857) compared to their White counterparts (Table 3). Similarly, Multiracial/Other respondents were also less likely to have career intentions in academia (OR 0.763, 95 CI 0.594–0.980) than White trainees (Table 3). There were no significant differences in academic, basic/translation, or clinical research intention between Hispanics and non-Hispanics. There were also no significant differences between Asians and Whites across all career intention comparisons.

**Table 3:** Logistic regression on career plan after residency, adjusting for training stage, sex, career sector intentions, specialty intentions, and mentorship status

	Effects of ethnicity on career intention, OR (95% CI)						
Career Plan	Asian or Pacific Islander vs. Black or African American *n* = 590	Asian or Pacific Islander vs. Black of Multiracial or Other *n* = 1166	Asian or Pacific Islander vs. White *n* = 3276	Black or African American vs. Multiracial or Other *n* = 742	Black or African American vs. White *n* = 3018	Multiracial or Other vs. White *n* = 3428	Hispanic vs. Not Hispanic *n* = 2945
Academia	**1.693 (1.031–2.781)**	1.100 (0.775–1.563)	0.840 (0.628–1.123)	0.650 (0.404–1.045)	**0.496 (0.322–0.764)**	**0.763 (0.594–0.980)**	1.087 (0.741–1.596)
Basic Research	**3.229 (1.123–9.281)**	1.475 (0.873–2.492)	1.013 (0.673–1.527)	0.457 (0.158–1.317)	**0.314 (0.115–0.857)**	0.687 (0.457–1.034)	1.289 (0.688–2.414)
Translational Research	1.113 (0.692–1.790)	0.922 (0.518–1.642)	1.372 (0.909–2.070)	**1.546 (1.107–2.159)**	1.488 (0.844–2.624)	**1.677 (1.004–2.799)**	1.126 (0.825–1.539)
Clinical Research	**1.292 (0.770–2.165)**	1.057 (0.750–1.489)	0.991 (0.749–1.312)	0.818 (0.495–1.352)	0.768 (0.485–1.215)	0.938 (0.731–1.203)	1.207 (0.704–1.499)

### Experienced and anticipated obstacles

Trainees of all ethnic backgrounds reported balancing family and work responsibilities as the top obstacle experienced during training, with balancing clinic, research, and education responsibilities as the second biggest challenge (Table 4). Black/African American trainees were most likely to describe experiencing discrimination and bias (18.6%). Rates of discrimination and bias as an experienced obstacle among Black/African American trainees ranged from 1.9 to 3.4-fold greater than trainees from other racial/ethnic groups.

**Table 4:** Career and non-career related responsibilities, obstacles, and factors in career selection

	Total n (%)	Asian or Pacific Islander n (%)	Black or African American n (%)	Multiracial or Other n (%)	White n (%)	***P***-value	Hispanic , n (%)	***P***-value (Hispanic)
Foreseeable non-work-related responsibilities DURING residency^1^ **n (% of 4433 Total)**								
Raising children	2573 (63.8%)	256 (60.0%)	104 (62.3%)	331 (57.4%)	1882 (65.8%)	**.0004**	142 (58.7%)	0.0990
Taking care of elderly parents	760 (18.8%)	130 (30.4%)	32 (19.2%)	166 (28.8%)	432 (15.1%)	**<.0001**	52 (21.5%)	0.3374
Being caretaker to others	562 (13.9%)	79 (18.5%)	31 (18.6%)	108 (18.7%)	344 (12.0%)	**<.0001**	49 (20.3%)	**0.0048**
Financial support of others	1095 (27.2%)	127 (29.7%)	75 (44.9%)	193 (33.5%)	700 (24.5%)	**<.0001**	84 (34.7%)	**0.0093**
Foreseeable non-work-related responsibilities AFTER residency^1^								
Raising children	3572 (88.6%)	373 (87.4%)	141 (84.4%)	508 (88.0%)	2550 (89.1%)	.2215	209 (86.4%)	0.2949
Taking care of elderly parents	2534 (65.0%)	326 (76.35%)	100 (59.9%)	409 (70.9%)	1788 (62.5%)	**<.0001**	143 (59.1%)	**0.0446**
Being caretaker to others	1224 (30.4%)	168 (39.3%)	51 (30.5%)	215 (37.3%)	790 (27.6%)	**<.0001**	75 (31.0%)	0.8877
Financial support of others	2152 (53.4%)	241 (56.4%)	101 (60.5%)	338 (58.6%)	1472 (51.4%)	**0.0014**	155 (64.1%)	**0.0008**
Experienced Obstacles^1^								
Balancing family and work responsibilities	1548 (38.4%)	154 (36.1%)	55 (32.9%)	195 (33.8%)	1144 (39.97%)	**0.0104**	109 (45.04%)	**0.0283**
Balance clinical, research, & education responsibilities	879 (21.8%)	106 (24.8%)	36 (21.6%)	129 (22.4%)	608 (21.2%)	.4039	60 (24.79%)	0.2425
Loan repayment	756 (18.8%)	78 (18.2%)	23 (13.8%)	90 (15.6%)	565 (19.7%)	**0.0386**	60 (24.8%)	**0.0138**
Lack of opportunity/funding	699 (17.3%)	81 (19.0%)	28 (16.8%)	105 (18.2%)	485 (17.0%)	.6972	56 (23.1%)	**0.0164**
Satisfactory professional development	375 (9.30%)	50 (11.7%)	17 (10.2%)	70 (12.1%)	283 (8.32%)	**.0079**	28 (11.57%)	0.2411
Under-compensation	263 (6.52%)	27 (6.32%)	5 (2.99%)	34 (5.89%)	197 (6.88%)	.2190	18 (7.44%)	0.5717
Discrimination/biases (gender/ethnicity)	276 (6.84%)	41 (9.60%)	31 (18.6%)	46 (7.97%)	158 (5.52%)	**<.0001**	22 (9.09%)	0.1779
Not finding position in desired location	318 (7.88%)	49 (11.5%)	8 (4.79%)	60 (10.4%)	201 (7.02%)	**0.0005**	11 (4.55%)	**0.0401**
Sexual harassment	49 (1.21%)	4 (0.94%)	2 (1.20%)	9 (1.56%)	34 (1.19%)	.8339	8 (3.31%)	**0.0028**
Malpractice/lawsuit	26 (0.64%)	1 (0.23%)	1 (0.60%)	4 (0.69%)	20 (0.70%)	.7337	1 (0.41%)	0.6018
Predicted Obstacles^1^ **n (% of 3819 Total)**						**<.0001**		0.0634
Balancing family and work responsibilities	1907 (49.9%)	194 (47.6%)	60 (38.7%)	252 (46.4%)	1401 (51.6%)		112 (47.46%)	
Balance clinical, research, & education responsibilities	225 (8.29%)	36 (8.82%)	18 (11.6%)	59 (10.9%)	225 (8.29%)		21 (8.90%)	
Not finding position in desired location	358 (9.37%)	51 (12.5%)	20 (12.90%)	67 (12.34%)	220 (8.11%)		16 (6.78%)	
Loan repayment	517 (13.5%)	26 (6.37%)	27 (17.4%)	51 (9.39%)	413 (15.2%)		38 (16.10%)	
Under-compensation	137 (3.59%)	12 (2.94%)	5 (3.23%)	20 (2.8%)	100 (3.69%)		13 (5.51%)	
Lack of opportunity/funding	322 (8.43%)	45 (11.0%)	7 (4.52%)	54 (9.94%)	216 (7.96%)		17 (7.20%)	
Malpractice/lawsuit	55 (1.44%)	6 (1.47%)	4 (2.58%)	6 (1.10%)	39 (1.44%)		3 (1.27%)	
Satisfactory professional development	98 (2.57%)	19 (4.66%)	5 (3.23%)	18 (3.31%)	56 (2.06%)		7 (2.97%)	
Discrimination/biases (gender/ethnicity)	44 (1.15%)	9 (2.21%)	7 (4.52%)	11 (2.03%)	17 (0.63%)		7 (2.97%)	
Sexual harassment	3 (0.08%)	1 (0.25%)	1 (0.65%)	0 (−-)	1 (0.04%)		1 (0.42%)	
Other	40 (1.05%)	9 (2.21%)	1 (0.65%)	5 (0.92%)	25 (0.92%)		1 (0.42%)	
Most important factors in career selection^3^ **n (% of 3833 Total)**						**0.0003**		
Ability to balance work & personal life	1371 (35.7%)	151 (36.7%)	52 (33.6%)	180 (32.9%)	988 (36.3%)		92 (39.32%)	
Opportunities for patient care	1303 (34.0%)	132 (32.0%)	46 (29.7%)	164 (30.0%)	961 (35.3%)		71 (30.34%)	
Financial security	160 (4.17%)	18 (4.73%)	9 (5.81%)	35 (6.40%)	98 (3.60%)		16 (6.84%)	
Opportunities to teach	101 (2.63%)	12 (2.91%)	3 (1.94%)	15 (2.74%)	71 (2.61%)		2 (0.85%	0.4831
Opportunities for research	414 (10.8%)	48 (11.7%)	12 (7.74%)	61 (11.2%)	293 (10.8%)		24 (10.26%)	
Opportunities for community service	122 (3.18%)	10 (2.43%)	13 (1.94%)	17 (3.11%)	82 (3.01%)		9 (3.85%)	
Opportunities for international work	109 (2.84%)	11 (2.67%)	11 (7.10%)	21 (3.84%)	66 (2.42%)		1 (0.43%)	
Autonomy	89 (2.32%)	14 (3.40%)	2 (1.29%)	20 (3.66%)	53 (1.95%)		5 (2.14%)	
Opportunities for student interactions	36 (0.94%)	1 (0.24%)	2 (1.29%)	6 (1.10%)	27 (0.99%)		3 (1.28%)	
Prestige	14 (0.36%)	1 (0.24%)	0 (−-)	4 (0.73%)	9 (0.33%)		1 (0.43%)	
Opportunities for travel	24 (0.63%)	3 (0.73%)	1 (0.65%)	4 (0.73%)	16 (0.59%)		1 (0.43%)	
Opportunities for local work	19 (0.50%)	1 (0.24%)	0 (−-)	7 (1.28%)	11 (0.40%)		1 (0.43%)	
Opportunities for national work	16 (0.42%)	4 (0.97%)	2 (1.29%)	2 (0.37%)	8 (0.29%)		1 (0.43%)	

^a^ Fisher’s Exact calculated due to minimum cell count violations

^1^ Respondents could select all applicable choices, will not sum to 100%

^2^ Respondents could select up to TWO choices, will not sum to 100%

^3^ Respondents could select up to THREE choices, will not sum to 100%

All trainees reported balancing family and work responsibilities as the top predicted obstacle (Table 4). Following this, Asian (12.5%) and Multiracial/Other (12.3%) indicated not finding a position in a desired location, whereas Black/African American (17.4%), Hispanic (16.1%), and White (15.2%) trainees selected loan repayment as the next most predicted obstacle. As with experienced obstacles, Black/African American trainees were also most likely to select discrimination and bias as a predicted obstacle, with a rate ranging from 1.5 to 7.2-fold greater than those of trainees of other races/ethnicities.

Whereas perceptions of research feasibility in surgical specialties did not significantly differ across racial/ethnic groups, perceptions of research feasibility in acute care medicine specialties were different (Table 5). In particular, 62.3% of White, 61.1% of Black/African American, 60.6% of Multiracial/Other, 60.1% of Hispanic, and 59% of Asian trainees viewed research-intensive careers in acute care medical specialties as difficult, highly difficult, or impossible. Conversely, Black/African American (7.6%) and Multiracial/Other (7.4%) trainees were most likely to view research intense careers in acute care medical specialties as highly feasible. In terms of feasibility of research intense careers in surgical specialties, there was a non-statistically significant trend toward fewer Black/African American trainees rating research intense careers in surgery to be difficult, highly difficult, or impossible (60.6%) as compared to Asian (64.8%), Multiracial/Other (62.8%), White (63.1%) and Hispanic (65.1%) trainees.

**Table 5:** Perceptions of research feasibility and the importance of mentoring

	Total, n (%)	Asian or Pacific Islander, n (%)	Black or African American, n (%)	Multiracial or Other, n (%)	White, n (%)	***P***-value	Hispanic, n (%)	Hispanic ***P***-value
How feasible is a research intense career in acute care medicine specialties?						**0.0315**		0.4490
Highly feasible	244 (6.33%)	18 (4.41%)	12 (7.64%)	41 (7.43%)	173 (6.32%)		15 (6.44%)	
Feasible	1234 (32.0%)	149 (36.5%)	49 (31.2%)	177 (32.1%)	859 (31.4%)		78 (33.48%)	
Difficult	1605 (41.6%)	176 (43.1%)	74 (47.1%)	209 (37.9%)	1146 (41.8%)		103 (44.21%)	
Highly difficult	711 (18.4%)	59 (14.5%)	22 (14.0%)	119 (21.6%)	511 (18.7%)		32 (13.73%	
Impossible	62 (1.61%)	6 (1.47%)	0 (−-)	6 (1.09%)	50 (1.83%)		5 (2.15%)	
TOTAL	3856 (100%)	408 (100%)	157 (100%)	552 (100%)	2739 (100%)		233 (100%)	
How feasible is a research intense career in surgical specialties?						0.0538		0.7247
Highly feasible	253 (6.56%)	30 (7.35%)	7 (4.46%)	48 (8.71%)	168 (6.13%)		17 (7.33%)	
Feasible	1169 (30.3%)	114 (27.9%)	55 (35.0%)	157 (28.5%)	843 (30.8%)		64 (27.59%)	
Difficult	1350 (35.0%)	140 (34.3%)	61 (38.9%)	168 (30.5%)	981 (35.8%)		79 (34.05%)	
Highly difficult	947 (24.6%)	107 (26.3%)	29 (18.5%)	157 (28.5%)	654 (23.9%)		61 (26.29%_	
Impossible	137 (3.55%)	17 (4.17%)	5 (3.18%)	21 (3.81%)	94 (3.43%)		11 (4.74%)	
TOTAL	3856 (100%)	408 (100%)	157 (100%)	551 (100%)	2704 (100%)		232 (100%_	
Can you currently identify a mentor(s) who has helped you progress toward &/or achieve your career goals?						0.1445		0.6488
Yes	2890 (76.3%)	322 (78.9%)	125 (82.2%)	409 (76.5%)	2034 (75.5%)		181 (77.7%)	
No	898 (23.7%)	86 (21.1%)	27 (17.8%)	126 (23.6%)	659 (24.5%)		52 (22.3%)	
TOTAL	3788 (100%)	408 (100%)	152 (100%)	535 (100%)	2693 (100%)		233 (100%)	
How important has mentorship been in your training thus far?						**<.0001**		0.3868
Very important	1274 (44.5%)	145 (45.6%)	72 (57.6%)	214 (52.5%)	843 (41.8%)		89 (50.3%)	
Somewhat important	1267 (44.2%)	129 (40.6%)	50 (40.0%)	159 (39.0%)	929 (46.1%)		68 (38.4%)	
Not very important	300 (10.5%)	42 (13.2%)	3 (2.40%)	32 (7.48%)	223 (11.1%)		18 (10.17%)	
Not at all important	25 (0.87%)	2 (0.63%)	0 (−-)	3 (0.74%)	20 (0.99%)		2 (1.13%)	
TOTAL	2866 (100%)	318 (100%)	125 (100%)	408 (100%)	2015 (100%)		177 (100%_	
How much importance is given to talents/accomplishments when recruiting applicants for jobs and/or positions in science and medicine?						**0.0405**		**<.0001**
A great deal of importance	1170 (30.9%)	152 (37.4%)	49 (32.0%)	174 (32.5%)	795 (29.5%)		70 (30.2%)	
A lot of importance	1835 (48.5%)	181 (44.5%)	66 (43.1%)	256 (47.9%)	1332 (49.5%)		110 (47.4%)	
Moderate amount of importance	719 (19.0%)	70 (17.2%)	36 (23.5%)	95 (17.8%)	518 (19.3%)		46 (19.8%)	
Little importance	60 (1.58%)	3 (0.74%)	2 (1.31%)	9 (1.68%)	46 (1.71%)		3 (1.29%)	
None at all	2 (0.05%)	1 (0.25%)	0 (−-)	1 (0.19%)	0 (−-)		3 (1.29%)	
TOTAL	3786 (100%)	407 (100%)	153 (100%)	535 (100%)	2691 (100%)		232 (100%)	
How much importance is given to connections/networking when recruiting applicants for jobs and/or positions in science and medicine?								**0.0053**
A great deal of importance	1223 (32.3%)	152 (37.3%)	67 (44.1%)	213 (39.8%)	791 (29.4%)		101 (43.2%)	
A lot of importance	1603 (42.3%)	157 (38.5%)	54 (35.5%)	205 (38.3%)	1187 (44.0%)		87 (37.2%)	
Moderate amount of importance	844 (22.3%)	86 (21.1%)	27 (17.8%)	96 (17.9%)	635 (23.6%)		43 (18.4%)	
Little importance	118 (3.11%)	12 (2.94%)	4 (2.63%)	21 (3.93%)	81 (3.01%)		3 (1.28%)	
TOTAL	3790 (100%)	408 (100%)	152 (100%)	535 (100%)	2695 (100%)		234 (100%)	

### Importance of mentoring and professional networking

The majority of trainees in each racial/ethnic group were able to identify a mentor who helped them progress toward and/or achieve their career goals (Table 5). However, perceptions in the value of mentorship varied between groups, with Black/African American trainees (97.6%) most likely to view mentorship as very or somewhat important in their training compared to other trainee groups (Asian 86.2%; Multiracial/Other 91.5%; White 87.9%; Hispanic 88.7%). Conversely, Asian trainees (13.8%) were most likely to describe mentors as being not very or at all important (Black/African American 2.4%; Multiracial/Other 8.2%; White 12.1%; Hispanic 11.3%) (Table 5).

More Asian trainees (81.9%) believed talents and accomplishments were given a great deal or a lot of importance in recruiting for jobs and positions in medicine compared to other groups (Black/African American 75.1%; Multiracial/Other 80.4%; White 79%; Hispanic 77.6%). Hispanic (80.4%) trainees were most likely to believe that connections and networking have a great deal of importance or a lot of importance when recruiting for jobs or positions in science and medicine, followed by Black/African American (79.6%), Multiracial/Other (78.1%), Asian (75.8%), and White (73.4%) trainees.

## Discussion

Previous studies investigating demographic disparities in the physician-scientist workforce have primarily focused on identifying disparate trends in the prevalence of underrepresented minorities in medical school/physician-scientist training program admissions, representation among academic faculty, or research grant application or funding success rates. While characterizing these statistical trends is important, efforts to improve demographic representation in the physician-scientist workforce would likely benefit from an understanding of the unique perspectives and challenges experienced by URMs early in training, such that these trainees can be effectively mentored throughout their training and career. In this regard, the present study represents the first in-depth survey of a nationally representative cohort of pre-doctoral physician-scientist trainees to evaluate differences in career aspirations, perceptions, and obstacles between those who identify as URM and their non-URM peers. Our results identify several differences in the training experience, interests, and intended career trajectory between URM and non-URM trainees, which may help to inform URM trainee recruitment and mentorship efforts to improve diversity in the physician-scientist workforce.

### Academic and research interests

One of the central goals of the study was to determine whether academic and research career interests vary according to trainee race/ethnic group. While we found that all racial/ethnic groups expressed a strong interest in pursuing a career in academia, the rates of academic interest did vary between groups, with Black/African American trainees reporting the lowest rates of interest in academia (40.8%). Importantly, racial/ethnic differences in academic career interests persisted after controlling for several demographic and trainee characteristics, including sex, age, training stage, access to mentors, specialty intention, and method of medical school funding by multivariate regression modeling. In particular, while there were no differences in academic career interests between Asian and White trainee cohorts, Asian trainees and White trainees were each independently significantly more likely than Black/American trainees to report interest in academic careers. In addition, White trainees were significantly more likely than Multiracial/Other trainees to express an interest in academic careers. These disparities in academic career interests at the predoctoral level are consistent with recent studies showing declining trends in URM representation among academic medicine faculty across various clinical specialties [17, 18].

With respect to research interests, our data suggest that there exist racial/ethnic differences in research career intentions. Among non-URM trainees, Asians demonstrated the strongest interest in basic/translational research (19.1%), while only 15.8% of White trainees shared this interest. Among URM trainees, there was a similar level of heterogeneity. While Black/African American respondents were least interested in basic/translational research (14.3%), Multiracial/Other (17.9%) and Hispanic (17.0%) trainees did express a substantial level of interest in this type of research. Similar to academic career interest, we found that after adjusting for multiple demographic and trainee covariates, Black/African American trainees remained significantly less likely than Asian and White trainees to report interest in basic research. However, it is interesting to note that Black/African American trainees did express a stronger interest in clinical research (8.4%) than other groups. These findings are important because previous studies have shown that participation in research during graduate medical training is significantly associated with full-time academic faculty appointment for URM trainees [19]. Together, this suggests that efforts to improve physician-scientist workforce diversity should include support and opportunities for URM physician-scientist trainees to engage in a broader range of research types from preclinical basic/translational research at the bench to patient centered research at the bedside.

### Value of professional activities: advocacy and international work

URM trainees were more likely to express an interest in incorporating advocacy and international work into their careers. This may reflect a desire by URM trainees to give back to their community, as seen in the high proportion of URM faculty who report working in areas that address health disparities [20]. However, this early desire for advocacy may also represent a source of burnout and dissatisfaction at the clinical or research faculty stage. Advocacy work may be transformed by what some have called the “minority tax” [21], or the additional burdens placed on URM faculty, from a career aspiration into an obligation which promotes attrition [22]. Though independent longitudinal validation of general URM interest in advocacy work is required, consideration of these activities in the academic promotion and tenure process could incentivize URM trainees with these interests to continue along this career trajectory and incorporate such work into an academic career.

### Training and career obstacles

The results from this survey identify key obstacles facing URM trainees at the predoctoral level. Concerning mechanisms for financing medical school, all racial/ethnic groups in this survey reported loans as the primary means of paying for medical school tuition. However, following loans, there were striking differences between trainees in different racial/ethnic groups. Whereas 24.3% of Asian, 23.3% of Multiracial/Other, and 13.5% of White trainees were dependent upon on family/partner support, only 2.5% of Black/African American and 5.8% of Hispanic trainees reported this as a primary means of paying for medical school. The limited familial/partner financial support among Black/African American and Hispanic trainees is consistent with previous reports demonstrating that these groups carry and anticipate substantially more educational debt than their peers [23, 24]. These financial concerns have also been shown to extend to the undergraduate level, underscoring the compounding nature of financial debt in these demographic groups prior to beginning medical school training [25, 26]. It has been suggested that perceived obstacles related to debt may subsequently influence specialty and career trajectories of these trainees, biasing trainees toward specialties and career practices with greater salaries and reimbursements [24, 27–30]. Although partial or full tuition scholarships are frequently offered by dual-degree programs, single-degree URM trainees interested in pursuing additional research training will likely disproportionately experience additional financial obstacles. The NIH Loan Repayment program (LRP) or other comparable funding mechanism could be leveraged to reduce this excess burden and encourage academic and research careers for single-degree URM physician-scientists [3, 31].

In addition to financial obstacles, perhaps most disturbingly, our survey demonstrates that Black/African American trainees reported experiencing significantly higher rates of discrimination and bias. These values ranged from 1.9 to 3.4-fold greater than trainees from other racial/ethnic groups. Several reports over the past few decades have noted that URM physicians report discrimination at significantly higher levels than White peers [32–34]. It is therefore disappointing and alarming that URM pre-doctoral trainees in this survey also reported experiencing such high rates of self-reported discrimination and bias at this stage in training, which is consistent with findings from other studies [35, 36]. These results are all the more concerning when placed against the urgent need to diversify the academic medical and physician-scientist workforce [37]. Medical school faculty frequently associate discrimination with low career satisfaction, and one factor behind the limited number of URMs in the physician-scientist workforce may be discrimination in the earliest phases of training [32].

### Mentorship

When exploring employment or other opportunities, non-URM trainees appeared to place more weight on talents and accomplishments, whereas URM trainees seemed to emphasize networking and connections. While URM trainees are more likely to have identified mentors, respondents did not indicate the nature of mentorship or the types of mentors (e.g. physician-scientists vs physicians), which could influence career choices. In this analysis, we also show that Black/African American trainees are less likely than trainees of other racial/ethnic groups to intend careers in academia and basic research. Owing to the extensive duration of training and the unique professional challenges associated with careers in academic medicine and basic research, perhaps one interpretation of these trends is that they reflect the dearth of available and accessible mentors in these career areas. Institutions and organizations aiming to improve diversity in physician-scientist training pipelines would do well to assign significant value to mentors and mentorship time for all trainees, including those who identify as URM.

### Limitations

This study has several limitations. First, the survey response rate was 27%, which is considerably lower than other physician-scientist trainee surveys conducted by single institutions or organizations such as the AAMC [38, 39]. One potential explanation for the relatively low response rate in this analysis is that we utilized an extensive 70-question survey to comprehensively assess trainee attitudes and perceptions. A second limitation is that the proportion of respondents identifying as Asian or Pacific Islanders in our survey was about half that expected from AAMC data, most likely reflecting oversampling of White and multiracial or other populations, as these both exceeded the expected proportions based on AAMC data. Another important limitation of this study is its cross-sectional nature, which cannot rule out the possibility of discordance between trainee survey responses and their ultimate career destinations. However, other studies have suggested that physician-scientist trainees tend to pursue careers in accordance with their training [40]. Finally, although this analysis identifies many pertinent factors and obstacles associated with URM trainee career goals, it does not provide insight into the reasons for why these factors exist among URM trainees. One important direction for future studies on this topic is to specifically understand why the career factors and obstacles identified here are uniquely prevalent in URM trainee cohorts. Such analyses are critical for optimizing policy and advocacy efforts aimed at recruiting and retaining URM trainees into the physician-scientist pipeline.

## Conclusions

A diverse physician-scientist workforce is critical for the future of academic biomedical research. Understanding the perspectives, experiences, and obstacles of URM physician-scientist trainees at the predoctoral level is an important step in efforts to diversify the physician-scientist workforce. In this cross-sectional analysis of over 4000 predoctoral physician-scientist trainees, we found that URM trainees do generally demonstrate an interest in pursuing academic and research activities in their career practices. However, URM trainees do differ from non-URM trainees in some career and specialty interests and in the importance and role of mentorship during training. These findings may serve to improve efforts to recruit and mentor the next generation of URM physician-scientists.

## Supplementary Information

**Additional file 1: Table S1.** Specialty intentions grouped by primary care, diagnostics, acute care and surgical specialties for our study participants. **Table S2.** Specialty intentions grouped by primary care, diagnostics, acute care and surgical specialties for the AAMC respondents. **Table S3.** AAMC respondents: Specialty choice by ethnicity. Supplemental data: survey tool.

**Additional file 2.**

## Data Availability

In accordance with IRB stipulations, data will not be posted online but can be made available at request.
