# Supplementary material for: Factors associated with underrepresented minority physician scientist trainee career choices
Source: BMC Med Educ. 2020 Nov 11;20:422. doi: 10.1186/s12909-020-02328-6 (PMC7656762; doi:10.1186/s12909-020-02328-6)
Supplement: Supplementary file 1 — Additional file 1: Table S1. Specialty intentions grouped by primary care, diagnostics, acute care and surgical specialties for our study participants. Table S2. Specialty intentions grouped by primary care, diagnostics, acute care and surgical specialties for the AAMC respondents. Table S3. AAMC respondents: Specialty choice by ethnicity. Supplemental data: survey tool. [file 12909_2020_2328_MOESM1_ESM.docx]

Supplementary Table 1 Our respondents

| **Specialty category** | **Total, n (%)** | **Asian or Pacific Islander, n (%)** | **Black or African American, n (%)** | **Multiracial or Other, n (%)** | **White, n (%)** | **Hispanic, n (%)** | **P-value** |
| --- | --- | --- | --- | --- | --- | --- | --- |
| Primary care/medicine | 1,485 (38.99%) | 153 (37.66) | 62 (39.78%) | 209 (37.79%) | 1,072 (39.4%) | 86 (37.38%) | p=0.001 |
| Surgical | 951 (24.79%) | 99 (24.39%) | 38 (23.74%) | 163 (29.47%) | 651 (23.91%) | 76 (33.03%) |  |
| Acute cate | 471 (12.27%) | 40 (9.85%) | 20 (12.82%) | 50 (9.04%) | 361 (13.26%) | 22 (9.56%) |  |
| Diagnostic | 651 (16.97%) | 66 (16.25%) | 28 (17.94%) | 99 (17.89%) | 458 (16.82%) | 29 (12.59%) |  |
| Undecided/other | 85 (2.21%) | 13 (3.2%) | 5 (3.21%) | 11 (1.99%) | 56 (2.06%) | 9 (3.91%) |  |

Supplementary Table 2 AAMC respondents

| **Specialty category** | **Total, n (%)** | **Asian or Pacific Islander, n (%)** | **Black or African American, n (%)** | **White, n (%)** | **Hispanic, n (%)** | **Native American, n (%)** | **Other, n (%)** | **P-value** |
| --- | --- | --- | --- | --- | --- | --- | --- | --- |
| Primary care/medicine | 1,230 (38.15%) | 284 (38.12%) | 66 (36.26%) | 765 (37.59%) | 82 (37.96%) | 13 (52.0%) | 19 (41.30%) | p=0.01 |
| Surgical | 866 (26.86%) | 182 (24.43%) | 49 (26.92%) | 536 (26.34%) | 87 (40.28%) | 6 (24.0%) | 7 (15.22%) |  |
| Acute cate | 479 (14.86%) | 100 (13.42%) | 34 (18.68%) | 299 (14.69%) | 38 (17.59%) | 3 (12.0%) | 5 (10.87%) |  |
| Diagnostic | 626 (19.42%) | 160 (21.48%) | 29 (15.93%) | 385 (18.92%) | 36 (16.67%) | 3 (12.0%) | 13 (28.26%) |  |
| Undecided/other | 69 (2.14%) | 19 (2.55%) | 4 (2.20%) | 44 (2.16%) | 0 (0.0%) | 0 (0.0%) | 2 (4.35%) |  |

Legend: Primary care/medicine includes family medicine, preventative medicine, pediatrics, medicine/pediatrics, palliative care, and internal medicine and most associated subspecialties. Surgical includes colorectal surgery, neurological surgery, obstetrics and gynecology, ophthalmology, orthopedic surgery, otolaryngology, plastic surgery, thoracic surgery, urology, general surgery, and vascular surgery. Acute care includes pulmonary critical care, emergency medicine, and anesthesiology. Diagnostic includes medical genetics, pathology, dermatology, radiology, nuclear medicine, radiation oncology, physical medicine and rehabilitation, psychiatry, neurology, and child neurology.

Supplemental Table 3 – AAMC Respondents

| Specialty | Asian n=744 | Black or African American n=182 | Hispanic n=216 | Native American n=25 | Pacific Islander n=1 | White n=2035 | Other  n = 46 | Pearson’s Chi-square |
| --- | --- | --- | --- | --- | --- | --- | --- | --- |
| Anesthesia | **63** | 13 | 17 | 2 | **0** | **112** | 3 | 0.065 |
| Dermatology | **25** | 4 | 4 | 0 | **0** | 65 | 2 | **0.031** |
| Emergency Medicine | 37 | 21 | 21 | **1** | 0 | **187** | 2 | 0.083 |
| Family Medicine | **27** | 10 | 23 | 3 | 0 | 148 | 5 | 0.066 |
| Medicine | **0** | 0 | 0 | 0 | 0 | 3 | 0 | **0.001** |
| Internal Medicine | **188** | 29 | 32 | 5 | 0 | 336 | 9 | 0.185 |
| Neurosurgery | **10** | 1 | 3 | 0 | 0 | 24 | 0 | **0.012** |
| Neurology | **21** | 4 | 7 | 1 | 0 | 57 | 3 | **0.029** |
| Obstetrics & Gynecology | **22** | 25 | 17 | 3 | 0 | 120 | 2 | 0.058 |
| Ophthalmology | **39** | 4 | 5 | 0 | 0 | 62 | 2 | **0.034** |
| Orthopedics | **23** | 4 | 7 | 1 | 0 | 111 | 0 | **0.045** |
| Ear Nose & Throat | **21** | 2 | 6 | 0 | 0 | 37 | 66 | **0.020** |
| Pathology | **19** | 1 | 3 | 0 | 0 | 40 | 1 | **0.020** |
| Pediatrics | **58** | 22 | 23 | 5 | 1 | 231 | 4 | **0.106** |
| Physical Medicine and Rehabilitation | **7** | 5 | 5 | 1 | 0 | 15 | 0 | **0.010** |
| Plastic Surgery | **7** | 0 | 31 | 0 | 0 | 20 | 0 | **0.009** |
| Preventive Medicine | **1** | 0 | 0 | 0 | 0 | 0 | 0 | **0.000** |
| Psychiatry | **25** | 6 | 11 | 1 | 0 | 91 | 4 | **0.042** |
| Radiology | **41** | 7 | 4 | 0 | 0 | 92 | 3 | **0.045** |
| Radiation Oncology | **22** | 2 | 2 | 0 | 0 | 25 | 0 | **0.016** |
| General Surgery | **40** | 7 | 10 | 0 | 0 | 126 | 2 | 0.057 |
| Thoracic Surgery | **2** | 2 | 3 | 1 | 0 | 9 | 0 | **0.005** |
| Urology | **17** | 4 | 5 | 1 | 0 | 27 | 1 | **0.017** |
| Medicine/Pediatrics | **10** | 5 | 4 | 0 | 0 | 47 | 1 | **0.021** |
| Other | **19** | 4 | 0 | 0 | 0 | 44 | 2 | **0.021** |
